# The application of implementation science theories for population health: A critical interpretive synthesis

**DOI:** 10.3934/publichealth.2018.1.13

**Published:** 2018-03-12

**Authors:** Jessie-Lee McIsaac, Grace Warner, Logan Lawrence, Robin Urquhart, Sheri Price, Jacqueline Gahagan, Mary McNally, Lois A Jackson

**Affiliations:** 1 Healthy Populations Institute, Dalhousie University, Canada; 2 Faculty of Education, Mount Saint Vincent University, Canada; 3 Faculty of Health, Dalhousie University, Canada; 4 Faculty of Medicine, Dalhousie University, Canada; 5 Faculty of Dentistry, Dalhousie University, Canada

**Keywords:** implementation science, population health, theory, framework, critical review

## Abstract

**Background and Purpose:**

Over the last decade, the field of implementation science (IS) has yielded an array of theoretical approaches to clarify and understand how factors influence the application and scaling-up of evidence-based practice in health care. These developments have led to questions about whether IS theories and frameworks might be of value to population health researchers and decision makers. The purpose of this research was to conduct a critical interpretive synthesis to explore, if, and how, key IS theories and frameworks might inform population health interventions aimed at reducing the burden of illness across populations.

**Methods:**

An initial list of theories and frameworks was developed based on previous published research and narrowed to focus on theories considered as formative for the field of IS. A standardized data extraction form was used to gather key features of the theories and critically appraise their relevance to population health interventions.

**Results:**

Ten theories were included in the review and six deemed most applicable to population health based on their consideration of broader contextual and system-level factors. The remaining four were determined to have less relevant components for population health due to their limited consideration of macro-level factors, often focusing on micro (individual) and meso (organizational) level factors.

**Conclusions:**

Theories and frameworks are important to guide the implementation and sustainability of population health interventions. The articulation of meso level factors common in IS theories may be of value to interventions targeted at the population level. However, some of the reviewed theories were limited in their consideration of broader contextual factors at the macro level (community, policy or societal). This critical interpretive synthesis also found that some theories lacked provision of practical guidance to address interventions targeting structural factors such as key social determinants of health (e.g., housing, income).

## 1. Introduction

Despite a wealth of clinical and health services research, there is a well-recognized *“failure to translate research into practice and policy”* [1]. This failure has created a growing and significant interest in the field of implementation science (IS) which is defined as “*the scientific study of methods to promote the systematic uptake of research findings and other evidence-based practices into routine practice, and hence, to improve the quality and effectiveness of health services”* [2]. Over the last decade, IS has yielded an array of theories and frameworks to help clarify which factors influence the implementation and sustainability of evidence-based practice in health care [3],[4]. Given the extensive theoretical work in the field of IS, we asked how theoretical developments in IS might be of value to population health intervention research (PHIR) which supports the development and uptake of evidence-based policies and programs aimed at improving population level conditions of risk and reducing health inequities. Our research, therefore, sought to explore, if, and how key IS theories and frameworks might inform the development, implementation and sustainability of population health interventions [5]. This is not to suggest that PHIR is atheoretical [6],[7]; but rather the intent was to explore if recent theoretical developments in the field of IS might be of value to researchers and decision makers working within population health.

### 1.1. Implementation science and population health

There are similarities across the fields of IS and population health. IS investigates the best approaches to move research into practice “*to improve the quality and effectiveness of health services”* [2] focusing mostly on changing healthcare professional and organizational behavior [2],[8],[9]. A population health approach focuses on improving the health of the entire population through action toward determinants that contribute to inequalities among population groups [10]. Examples of population health interventions include affordable housing policies, immunization programs, and tobacco taxation, and PHIR aims to contribute relevant, credible, and timely evidence to enable decision makers to develop and implement policies and programs at the population-level [5]. In both, success in achieving intended impacts relies on evidence that is generated through meaningful and explicit engagement with the population of interest (e.g., patients/community) and the recognition of the importance of context to ensure interventions are relevant for individuals/patients, providers/practitioners, organizations and communities [11],[12]. Further, just as IS supports the need to develop and draw from relevant theories and theoretical constructs, PHIR supports the application of appropriate theories to ensure that evidence-based interventions reach whole populations/communities [13],[14]. Population health interventions are often directed at large groups or populations, targeting large-scale policies or entire communities, and focus on social-cultural factors and environmental conditions [5],[15]. Interventions that are aimed specifically at improving the social determinants of health are also often fundamental considerations in a population health approach [12],[16], including interventions that target key determinants such as income, housing, social exclusion, and food insecurity [17]. Considering these similarities and differences between IS and population health, the purpose of this synthesis was to critically assess the potential relevance of existing theories and frameworks developed in the field of IS to population health interventions.

## 2. Methods

**Figure 1.**
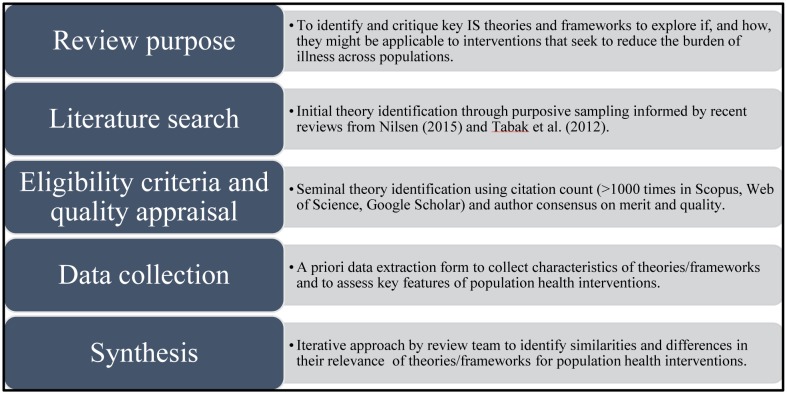
Overview of critical interpretive synthesis approach.

A critical interpretive synthesis approach was used to identify key IS theories and frameworks, and to explore if, and how, they might be applicable to population health interventions [18]. For the purposes of this research, “theories” were defined as a set of analytical principles or statements with explanatory power [3],[19],[20], and “frameworks” as a structure without explanatory power that includes descriptive categories outlining various constructs that may explain the relations between them [3],[21]. The steps outlined in a recent scoping review of knowledge synthesis methods [18] were used to inform the operationalization of the review (see Figure 1).

The critical interpretive synthesis method offered an iterative approach to refine the research question, search and select articles, and define and apply codes and categories [18]. A core review team (JLM, GW, LL, RU, and a research assistant) conducted the primary review of relevant theories, and the larger authorship team (SP, JG, MM, LJ) reviewed the analysis, contributed feedback to preliminary results and revised the manuscript.

A number of strategies were used to collect literature on IS theories and frameworks, which is supported by the emergent and exploratory nature of the review method [18]. Drawing on existing comprehensive research, the search began with an examination of two recent reviews of IS theoretical approaches that provided a comprehensive synthesis of current IS theories and frameworks [3],[4]. An initial list of theories was developed using the more recent review by Nilsen [3] using their categories of “determinant frameworks” and “implementation theories” as these categories emphasized theoretical explanations to implementation. To validate and expand the initial list, theories categorized as having a focus on “implementation” (i.e., putting evidence-based interventions into practice/policy) in the review by Tabak et al. [4] were also included. These theories were determined to be relevant for this review due to their focus on theoretical constructs to inform implementation.

Thirty-one theories and frameworks were initially identified. Theories cited by others more than 1000 times in three bibliographic databases (Scopus, Web of Science, Google Scholar) provided the basis for further narrowing the list to those considered “seminal” or “formative” for the field of IS. Ten theories and frameworks were identified for inclusion through this process, and consensus on their strength of conceptual support for implementation and merit for the review was obtained from the authorship team.

A standardized data extraction form was developed and used to identify theory development and evolution, key theoretical components and mechanisms, and other descriptive features for each of the ten theories (Supplementary Table). The theories were also assessed using a data extraction process developed a priori to appraise if and how they might inform PHIR. The data extraction process was guided by three questions, and coding definitions (see Table 1).

Data were extracted by the core review team, with at least two reviewers supporting extraction and verification of each theory. The review team appraised the resulting data extraction table following the secondary review to ensure consistency in the results. The information extracted enabled the review and authorship teams to critically interpret the results, in an iterative manner, by identifying emerging similarities and differences across the theories and frameworks in terms of their relevance to inform population health interventions.

Although not the main focus of this study, a secondary search within the published literature was completed to further understand where the final 10 theories/frameworks to population health were being utilized. Using each theory's foundational paper and applying population health search terms based on the Population Health Search Strategy developed by the United States National Library of Medicine [23], a search of relevant bibliographic databases (MEDLINE, Embase, CINAHL, Web of Science) was conducted. The results were screened by two reviewers (JLM, MM); examples of theories applied to population health research were identified (see Table 2).

**Table 1.** Data extraction questions, focus and coding developed for the review.

Key questions	Could the theory help to inform interventions seeking to address populations (e.g., change conditions of risk?)	Could the theory help to inform interventions seeking to address social determinants of health?	Could application of the theory generate evidence to inform policy/practice change at a population level?
Key focus	Population health provides insights into the needs of populations, rather than the needs of individuals [22]. Population health interventions are intended to change the social-cultural and environmental conditions of risk to support the overall health of populations and reduce health inequities [5],[15].	The social determinants of health influence the health of populations. Although variously defined, one definition includes income and social status; social support networks; education; employment/working conditions; social environments; physical environments; personal health practices and coping skills; healthy child development; gender; and culture [16].	Population health research is aimed at generating relevant, contextually sensitive, credible and timely knowledge to enable decision makers to use evidence to improve policies and programs that prevent disease at a population level [5].
Coding definitions	Yes, if macro (e.g., societal, policy) and community-level factors are considered, including a focus on populations and systems. Possibly, if remains focused on more micro (e.g., individual) or organizational levels.	Yes, if social determinants of health are considered and integrated into components of the theory/framework. Possibly, if social determinants of health are not explicitly considered in the components of the theory/framework.	Yes, if application of theory/framework could result in useful information to inform policy/practice change. Possibly, if application of theory/framework focused more on research utilization and does not seem likely to be useful for policy/practice change.

## 3. Results

A comprehensive description of the development of all ten theories is available in the Supplementary Table; the results of the critical appraisal are available in Table 2.

**Table 2.** Results of the critical interpretive synthesis based on a priori data extraction criteria.

Theory	Could the theory help to inform interventions seeking to address populations (e.g., change conditions of risk?)	Could the theory help to inform interventions seeking to address social determinants of health?	Could application of the theory generate evidence to inform policy/practice change at population level?	Examples of theory application in population health identified through secondary review.
Absorptive Capacity (ACAP) (Zahra & George, 2002)	Possibly; remains focused on organizational level. Does explore larger systems factors, with the exception of structural, behavioural or political “social integration mechanisms”.	Possibly; could potentially be taken into consideration upon application of the theory through the activation triggers and social integration mechanisms.	Yes; could provide information on what is contributing to the success of an organization and what can improve its success.	One identified related to knowledge brokering in the health sector [24].
Active Implementation Framework (Fixsen et al. 2005)	Possibly; consistent focus on the community level but does not fully consider populations or systems.	Possibly; could be applied to implementation in areas that address SDH (e.g. education).	Yes; but the framework focuses more on community-level implementation.	Several identified related to sexual health [25], school-based positive behavioral supports [26], and HIV [27].
Consolidated Framework for Implementation Research (Damschroder et al. 2009)	Yes; approaches implementation through a multilevel lens and recognizes factors existing at multiple levels of the system.	Possibly; does consider the importance of the organization knowing and prioritizing patient needs	Yes; could help to explore practice and policy change as it relates to implementing new programs.	Many identified related to various population health issues, such as tobacco cessation [28], child mental health [29] and nutrition [30], oral health [31], HPV vaccine [32].
Diffusion of Innovations for Service Organizations (Greenhalgh et al. 2004)	Yes; although focused primarily at the organizational level, considers system-level factors that affect diffusion, dissemination and implementation of innovations.	Possibly; sub-factors are associated with the innovation so could be adapted to address SDH.	Yes; provides a wide variety of factors that could be explored to generate evidence for practice and policy change.	Two identified related to HIV testing [33] and public health policy [34].
Ecological Framework (Durlak & Dupre, 2008)	Yes; takes a multilevel ecological approach by considering individuals in the context of their environments.	Yes; characteristics of the innovation consider community needs, values and cultural norms and examples of SDH interventions provided.	Yes; relevant factors described that could become levers for changes to policy and practice.	None identified.
Implementation Effectiveness Model (Klein & Sorra, 1996)	Possibly; focused at organizational level and does not explicitly address populations.	Possibly; does consider how climate within an organization influences implementation.	Possibly; it may generate evidence at the organizational level, this would not be widely applicable at a population level.	One identified related to mental health amongst low-income women and health care practitioners [35].
Multilevel Change Framework (Ferlie & Shortell, 2001)	Yes; multi-level and highlights the importance of the legal, political, and economic environment.	Possibly; however, factors focused on health care quality.	Yes; system-wide changes are considered in the scope of multilevel change and could provide useful evidence to inform practice/policy change.	Two identified related to HIV testing [36] and quality improvement in public health [37].
Promoting Action on Research Implementation in Health Services (Kitson et al., 1998)	Possibly; conceived as an organizational framework for health care and does not explicitly address populations.	Possibly; SDH could be considered as part of the context where the evidence is being implemented.	Yes; could generate evidence to inform practice change within particular settings but may have fewer implications for policy change.	Several identified but mostly related to health care settings: Oral health in home care setting [38]; community-based mental health care [39]; community-based health for people with disabilities [40].
Sticky Knowledge (Szulanski, 1996).	Possibly; focused more on the organizational level, but could be adapted to help inform the transfer of knowledge to populations.	Possibly; developed within a relatively narrowly-defined organizational context.	Yes; could be used to identify different barriers to implementing policies/programs, but developed in a business context may limit transferability to a larger or more diverse setting.	None identified.
Theoretical Domains Framework (Michie et al., 2005) & Behaviour Change Wheel (Michie et al., 2011).	Yes; although TDF remains largely focused on individuals; the policy categories of the BCW provide an explicit focus at the level of populations.	Possibly; the TDF does consider the role of resources and environment on behaviour change and the policy categories of the BCW could help to focus interventions relevant to SDH.	Yes; use of BCW could inform policy and practice, but the focus of the TDF on practice change suggests its influence on policy may be limited.	Many identified related to a various population health issues, such as tobacco cessation [41], childhood nutrition [42] and physical activity [43], oral health [44].

None of the theories provided an explicit or comprehensive framework for how to address population health interventions. However, six theories had underlying constructs/principles that were considered applicable to population health: The Ecological Framework [45], the Active Implementation Framework [46], Diffusion of Innovations for Service Organizations [47], Multi-level Change Framework [48], the Consolidated Framework for Implementation Research [49], and the Theoretical Domains Framework/Behaviour Change Wheel [50],[51]. These six theories considered influences beyond the organizational level, such as macro or system-level contexts (e.g., societal, policy and community factors) that are relevant for interventions targeting populations. Further, although none of these theories explicitly referred to key social determinants of health or population-level policies and practices, their identified constructs could be of value to PHIR. In comparison, the remaining four theories appear to require significant adaptation or expansion to be applicable to population health given their emphasis on the organizational level within healthcare services (Promoting Action on Research Implementation in Health Services framework [52]) or the business/management sector (Absorptive Capacity [53], Implementation Effectiveness Model [54] and Sticky Knowledge [55]). Although organizational level considerations are an important component of population health, these frameworks did not provide explicit consideration of broader community, policy or societal factors that play central roles in population health interventions.

### 3.1. Theories appraised as relevant for population health

Durlak and DuPre's Ecological Framework was identified as one theory that did, to some extent, refer to populations and changing the conditions of risk, which is likely due to its origin in community-based health promotion and disease/substance prevention settings [45]. Based on their review of 542 relevant studies (including 5 meta-analyses), the authors concluded that five categories of influencing factors are critical to implementation, and provide detail about these factors. The central two categories are the innovation delivery system (e.g., organizational capacity), and the innovation support system (e.g., training, technical assistance), which represent an organization's capacity for effective implementation. Their ecological lens posits that successful implementation is dependent on factors in three other categories: Innovation characteristics (e.g., compatibility with existing practice, adaptability to new contexts or needs); characteristics of those providing the innovation (e.g., perceived need, benefit, delivery skills); broader community factors (e.g., current state of the research, available funding, policies, politics/ideological climate). These five categories are interrelated and were also dependent and may change based on different local contexts. The Ecological Framework provides relevant core components for population health that could be helpful in generating evidence for policy and practice change (e.g., guidance for strengthening intervention delivery and support systems). Also the Ecological Framework constructs were similar to constructs identified in other IS theories in this review, including factors such as training [46],[47], managerial support, shared vision and organization norms regarding change [47]. However, there were limitations noted as the framework was not based on a systematic review of the literature, nor have its components been subsequently tested and refined. There were no examples of the Ecological Framework applied to population health research identified through the secondary review of literature.

The Active Implementation Framework [46] considers evidence from multiple fields with the aim of supporting interventions related to mental health, social services, justice, education and early childhood. The importance of community involvement and assessing community readiness for change is a focus of the theory, which is detailed in the framework's essential components (source, destination, communication link, feedback mechanism, sphere of influence) and relevant for population health interventions. However, the authors' reference to essential implementation outcomes remains focused on changes in individual behavior, relationships and organizational structure. Although these outcomes are sometimes important to changing the conditions of risk in a population, the emphasis on individual and organizational characteristics may limit its applicability to population-level change. There is some recognition of system-level partnerships and the stages of implementation that suggest appropriate times to deliver interventions, but the Active Implementation Framework would require further details on the operationalization of the components if it was to inform implementation of evidence into policy and practice, particularly among changing population-level circumstances with dynamic or unsupportive “sphere of influences” (e.g., social, economic or political factors that influence people, organizations and systems). There were several applications of this framework identified to population health interventions [25]–[27] in the secondary review of the literature.

The four additional theories deemed as relevant for population health originated from health services and care delivery but their recognition of external influences and explanation for implementation offer important insights for population health. For example, Greenhalgh and colleague's framework for Diffusion of Innovations for Service Organizations is focused primarily at health service organizations yet its comprehensive description of factors and determinants relevant for the development, coordination and delivery of interventions offers insight into how an intervention might achieve broader population level change through its assimilation into larger systems [47]. The “Outer Context” domain in this theory would also be relevant for large-scale initiatives in population health (e.g., sociopolitical climate and interorganizational networks) and the domain of “System Readiness for Change” could help to articulate readiness for change across communities or populations. Decision makers could also use information generated from this theory to identify/evaluate factors that affect the spread of population health interventions, but taking into account the multitude of factors, it may be complex for decision makers to operationalize this framework in a way that informs the generation of evidence for policy and practice change. Congruently, there were only two examples identified of the application of this theory in population health [33].

The Multilevel Change Framework [48] focuses on quality improvement in health care by advancing previous work [56] and captures multiple levels of change for improving quality (individual, groups/team, organization, larger system/environment). In an effort to identify factors that might influence adaptation and associated properties, the resulting framework includes essential core properties to support change, such as leadership, organizational culture, microsystem/team development, information technology to inform continuous improvement and accountability. The emphasis on intervening, or at least considering, all levels of change in interventions suggest that it could provide useful information to inform population-level policy and practice, especially as it considers the impact of legal, political and economic environments. However, although the authors examined various quality strategies to develop the framework, the review methods were not systematic which may limit the comprehensiveness of factors influencing implementation. This theory was not commonly applied in the population health literature, with only two examples identified through the secondary search [36],[37].

The Consolidated Framework for Implementation Research (CFIR) was create to synthesize existing implementation theories such as Greenhalgh et al. [47],[57] framework as well as eighteen other models and frameworks. CFIR conceptualizes implementation from a multilevel lens. The framework identifies influences from “Outer” and “Inner Settings” that consider external policy and cultural factors, acknowledging that these factors exist at multiple levels of a system. CFIR also considers the importance of socio-political conditions or circumstances that can influence implementation. This is relevant to the context of population health (e.g., nature and quality of social networks both inside and outside the system, governmental policy and regulations) and could help to inform an understanding of the reasons for successful policy and practice change initiatives [49]. However, it was noted that the explanations for these various external/system factors were somewhat superficial, suggesting that further clarification is needed to inform its usefulness in population health. One example of this is the construct “Patient Needs & Resources” in the “Outer Setting” domain. This construct could be clarified and expanded to represent a more inclusive definition of the population-level influences on implementation processes, including the values, preferences, and circumstances of individuals within communities. The secondary literature review identified numerous examples of the application of CFIR to a range of population health interventions (e.g., [28]–[32]).

Finally, although originating from health psychology and using the lens of individual behavior change, the Theoretical Domains Framework (TDF) [50] and the subsequent development of the Behavior Change Wheel (BCW) [51],[58] provides relevant reflections for population health interventions when considered as interconnected theories (i.e., considering the BCW as an extension of TDF). On its own, the TDF is used to identify individual-level determinants that impede or support individual behavior change, although the domains of “Social Influences” and “Environmental Context and Resources” are relevant for population health research. The strength of the TDF/BCW lies in their combined use: When the population health issue is viewed as a behavioral issue (e.g., individuals within a particular population not seeking cancer screening), the TDF can guide a needs assessment to identify the main barriers to change. Once barriers are identified, they are situated within the “COM-B” (Capability, Opportunity, Motivation, Behavior) system in the centre of the BCW. Depending on where the barriers lie, they can then be linked to evidence-based “Intervention Functions” (e.g., education, persuasion, training, environmental restructuring) within the BCW, which is based on previous research on addictions [59]. In turn, these intervention functions can be linked to specific “Policy Categories” of the BCW (e.g., fiscal measures, social planning, legislation), making it the only theory we reviewed that provided clear guidance on how to target individual-level health behaviors at the population level. Thus, information generated by use of the BCW could be used to inform population level policy and practice through application of “Intervention Functions” and “Policy Categories”. The secondary literature review identified numerous population health interventions that applied this framework [41]–[44].

### 3.2. Theories appraised as requiring adaptation to inform population health

The remaining four theories included in the review were determined to have less relevant components for population health and would require significant adaptations or expansions [52]–[55]. Common among these theories was their limited consideration of macro-level factors, often focusing only at the organizational level. Although organizational-level influences are important considerations for population health programs, it is important that these are considered in the broader context of communities and populations. Similar to the theories described in the previous section, the social determinants of health were not specifically considered.

The Promoting Action on Research Implementation in Health Services framework [52] was conceived as an organizational framework in health care, and as a result the core components of “Evidence”, “Context” and “Facilitation” remain tightly focused on health services organizational settings, making it unclear how the framework might be of value for interventions targeting populations. Absorptive Capacity [53], Implementation Effectiveness Model [54] and Sticky Knowledge [55] were all developed from the field of management and primarily focused on organizational change. These four frameworks may be able to generate evidence to inform practice change within particular settings (e.g., determinants of implementation and what leaders/teams might do to facilitate implementation in their contexts), but they appear to have fewer implications for policy change. Components of these theories could be adapted to improve their relevancy; for example, although the definition of context in Sticky Knowledge referred more to organizational characteristics, this could be adapted for contextual considerations at the population level [55]. Application of these theories could generate evidence to inform change at the organizational level (e.g., considerations of “Competitive Advantage” from Absorptive Capacity [53] and “Innovation/Implementation Effectiveness” from the Implementation Effectiveness Model [54], but as they were developed from a business/management perspective, these factors may not be widely applicable at a broad population health level. The secondary review of literature identified few examples of the application of these theories to inform population health intervention. One example was identified from both Absorptive Capacity [24] and Implementation Effectiveness [35] and several from the Promoting Action on Research Implementation in Health Services framework, although these latter examples were primarily oriented toward health care [38]–[40].

## 4. Discussion

Given the growing burden of chronic and infectious diseases globally, there is a need for researchers and decision makers to identify effective and efficient interventions that can be scaled-up improve population health. Theories and frameworks can help guide population health interventions by providing a comprehensive explanation of how multi-level factors affect successful implementation. The purpose of this review was to explore if and how key IS theories and frameworks might be applicable to interventions aimed at improving population level conditions of risk and reducing health inequities. It is evident from our findings that IS theories and frameworks could help to identify potential constructs or components to inform implementation strategies for population health interventions. However, there were some limitations to their application when considering the importance of factors beyond the organizational level, particularly at the macro level which is a level that often influences policies and programs targeting populations.

Broadly defined, context shapes research utilization in policy and program development [60] and is relevant to both IS and PHIR. Although all of the reviewed theories/frameworks identified various contextual elements as being important, they often limited their focus to the organizational-level and did not provide a comprehensive description of the community, and socio-cultural and political contexts that need to be considered in PHIR. Population health interventions need to not only consider the socio-demographic and health characteristics of the population (e.g., age, disease status, education level) but also the characteristics of communities (e.g., rurality, culture, resource availability), as these can all affect implementation and sustainability. The potential levels targeted by the intervention are also important for various reasons not the least of which is because it influences how researchers work with relevant stakeholders to design, implement, evaluate and scale-up evidence-based interventions [61]. For example, community-level characteristics may also be relevant for some health service interventions such as primary healthcare, but these factors are a core consideration in population health interventions to ensure relevancy to the targeted population. Several reviewed theories/frameworks provided some considerations for interventions at a broader community-level, including specific mention of factors at this level in Durlak and Durpre's framework through politics, funding and policy. This could be used to consider how, for example, funding for public health interventions shapes the feasibility of implementation strategies (e.g., was the reason for deciding on a given implementation strategy guided primarily by budget constraints at the expense of effectiveness?). Other theories/frameworks deemed as relevant also included some mention of community-level intervention, such as CFIR's “outer” and “inter” constructs (e.g., external policies and incentives, culture) and the policy categories in the BCW (e.g., fiscal measures, legislation) but the information was not as detailed. Similarly, these external factors can help explore implementation strategies, such as how closely they align with existing external policies (CFIR) or influenced by legislative changes which may or may not align with current practices (BCW). Additional clarification of community-level and macro-level factors would be important adaptations to the reviewed IS theories/frameworks to inform population-level interventions. In PHIR there is a focus on understanding how social, economic, political, and environmental conditions influence the health of a community or population and effective interventions should address multiple levels at the same time [5],[10]. This multi-level planning that is required to simultaneously target micro-, meso-, and macro-levels was not fully addressed in the reviewed IS theories/frameworks, although most did acknowledge the complexity of interventions.

Despite their potential theoretical insight to population health, we identified several theories that were not commonly applied to population health interventions, which may be a result of their broad nature (e.g., Durlak and Dupre's Ecological Framework, Multilevel Change Framework) or the multitude of complex factors in the frameworks may present pragmatic limitations (e.g., Greenhalgh et al. Diffusion of Innovation). In comparison, the theories that were more commonly applied in the population health literature were those that built upon previous theories and also seemed to have investigator teams promoting its use through websites, books and publications (e.g., CFIR, TDF and BCW and Promoting Action on Research Implementation in Health Services framework). A recent study explored the use of IS theories in the field and reported that implementation scientists use various criteria to select theories, but suggested that the process may be informed by convenience or prior exposure [62]. Future research is needed to explore uses of IS theories and frameworks in PHIR to identify the reasoning of their use, how they have been adapted and applied in different population-level contexts. It is also important to note, that Durlak and Dupre's Ecological Framework was the only theory/framework to explicitly include factors relevant to the social determinants of health and provide relevant examples of social policy interventions that would address these concerns. Further examination of the theories/frameworks and their application could help to identify further constructs relevant to the social determinants of health that were less explicitly defined in the source documents.

There were numerous similarities across the reviewed IS theories and frameworks, likely a result of the developing nature of the field. Many of the theories/frameworks built upon prior work. For example, inherent in its name the *Consolidated* Framework for Implementation Research [49] brought together learnings from previous theories, including four of those explored in this review [46],[47],[52],[54]. Another example is the extension of the TDF to the BCW, representing the development and refinement of behavior change research over a decade. The more recent BCW provides explicit guidance in intervention design that can bridge the gap between individual health behaviors and large-scale intervention strategies [50],[51]. There was also a clear difference between factors identified as relevant for implementation, depending on the field the theories and frameworks originated from. For example, those developed in business settings are more mechanistic in that they did not account for external factors which might make implementation difficult, and thus are less useful for PHIR [53]–[55]. This does not mean that these theories cannot lend themselves to population health interventions; conversely, it is possible that their focus on organizational-level factors might provide added depth or insight that broader frameworks or theories do not. However, based on the results of this review, we feel that the absence of external factors is a critical gap which makes them less suitable for use in population health interventions, and that alone they are less relevant than other IS theories or frameworks that do not account for external factors. In contrast, most of the reviewed theories from a health care context acknowledged multiple levels of action [47]–[50],[58] or at least contextual factors [46],[52]. Moreover, three theories/frameworks that we identified to be useful to PHIR [45],[47],[49] agreed on the importance of five factors when assessing implementation viability which might offer potential value to PHIR, including: (1) innovation characteristics; (2) individuals involved with its implementation; (3) organizational capacity to support implementation; (4) broader-level policy/politics/financial/social contexts; (5) the implementation process.

The overlap of important factors across theories and frameworks may limit the operational use to a “checklist” of factors to consider in implementation research. This is likely due to the complexity of implementation in health settings, as the theories/frameworks that were deemed less useful to PHIR were also the ones that provided more details on the operationalization of theoretical components [53]. Unfortunately, beyond indicating the relative importance of factors related to effective implementation, few theories provided direction on how the components of these different frameworks might work together, or if they need to be applied as a whole theory/framework. The CFIR is sole theory that specified it is meant to be a menu of constructs the researcher chooses from based on the salience of the construct to the context, so it is not necessary it be applied as a whole model [49]. This “menu” approach presents an additional challenge for researchers who cannot target all relevant factors: How will constructs be prioritized and selected? Tools such as the APEASE criteria (Affordability, Practicality, Effectiveness, Acceptability, Safety, Equity) [58] or worksheets developed by Flottorp and colleagues [63] offer guidance on prioritization and assessment of factors within an implementation strategy. These tools enhance understanding of the implementation context(s), which the interventionist can use to identify what factors are critical to address. This concept of “critical capacities” has been noted in the political science literature; many factors may contribute to success in complex, dynamic systems (e.g., population health interventions, federal policy making), but not at all times or across all conditions [64]. It also aligns with realist philosophy that supposes that different contexts can leverages aspects of interventions to make them successful [65].

### 4.1. Strengths and limitations

One of the strengths of this review is that the authors involved with this paper provided varying perspectives due to their diverse backgrounds (e.g., public health, health promotion, population health, health services research, implementation science), which provided safeguards for potential disciplinary biases in analytic approaches. An additional level of rigor was added by having a larger authorship team review and provide feedback on nascent findings. It is important to note the review team broadly interpreted the terms used in the frameworks and theories as many of the terms were specific to health care settings (e.g,. patients/clients could also refer to community members, or providers could refer to practitioners). However, it was acknowledged that this broad interpretation of terms may not be relevant or applicable to certain communities or populations. Several limitations to the review should be noted, including the fact that full transparency of the review procedures are not possible due to the interpretive nature of the process [18]. However, the various steps taken by the team to ensure rigour were used to frame the analysis have been explained. A second limitation is that the review only included seminal theories; this process was deemed necessary to keep the review focused and manageable yet as a result, we may have missed relevant theories that were not as widely cited in the literature.

## 5. Conclusions

Theories and frameworks are important to help clarify factors influencing the implementation and sustainability of evidence-based population health interventions. This study explored how key theories from the field of IS might inform policies and programs seeking to reduce the burden of illness across populations. The theories most relevant to PHIR were expansive frameworks that considered macro-level contexts, but these theories were still limited in their practical guidance to inform population level interventions. A plethora of constructs were offered by the reviewed theories and frameworks as factors to consider or assess in implementation, with five categories of factors common appearing throughout all theories: (1) innovation characteristics; (2) individuals involved with its implementation; (3) organizational capacity to support implementation; (4) broader-level policy/politics/financial/social contexts; (5) the implementation process. However, there was limited explanation of how they might be of practical value to address structural factors such as the social determinants of health.
