# Supplementary material for: The application of implementation science theories for population health: A critical interpretive synthesis
Source: AIMS Public Health. 2018 Mar 12;5(1):13–30. doi: 10.3934/publichealth.2018.1.13 (PMC6070464; doi:10.3934/publichealth.2018.1.13)
Supplement: Supplementary file 1 [file publichealth-05-01-013-s01.pdf]

AIMS Public Health

AIMS Public Health, 5(1): 13–30.
DOI: 10.3934/publichealth.2018.1.13
Received: 18 November 2017
Accepted: 07 February 2018
Published: 12 March 2018

http://www.aimspress.com/journal/aimsph

Review

The application of implementation science theories for population health: A critical interpretive synthesis

Jessie-Lee McIsaac[1,2,*], Grace Warner[1,3], Logan Lawrence[1,3], Robin Urquhart[1,4], Sheri Price[1,3], Jacqueline Gahagan[1,3], Mary McNally[1,4,5] and Lois A Jackson[1,3]

[1] Healthy Populations Institute, Dalhousie University, Canada
[2] Faculty of Education, Mount Saint Vincent University, Canada
[3] Faculty of Health, Dalhousie University, Canada
[4] Faculty of Medicine, Dalhousie University, Canada
[5] Faculty of Dentistry, Dalhousie University, Canada

* **Correspondence:** Email: Jessie-Lee.McIsaac@msvu.ca; Tel: +9024576508.

Supplementary

Table S1. Final theories, their development and components.

	1.) Field<br>2.) Type of theory/framework<br>3.) Level of focus[1]	Focus of theory/framework	Approach to theory/framework development	Development or evolution of theory/framework	Core components and sub-components of theory/framework
Absorptive Capacity (ACAP) (Zahra & George, 2002)	1.) Management<br>2.) Implementation theory<br>3.) Organizational	To examine the creation and utilization of knowledge by businesses in order to gain and sustain a competitive advantage.	Authors conducted a non-systematic review of the literature of conceptual and empirical studies using ACAP. No review methods were outlined. Authors demonstrated that over time researchers have studied ACAP at different levels of analysis and adopted multiple measures of the construct. Authors proposed a reconceptualization of ACAP.	Informed by[2]:<br>- Cohen and Levinthal (1990)<br>- Various other studies also noted<br>Further development by[3]:<br>- Zahra & George (2002) reviewed the framework and proposed a reconceptualization.	Components[4]:<br>- Potential capacity (PACAP)<br>- Realized capacity (RACAP)<br>Dimensions:<br>PACAP:<br>1.) Knowledge acquisition<br>2.) Knowledge assimilation<br>RACAP:<br>3.) Knowledge transformation<br>4.) Knowledge exploitation<br>PACAP and RACAP build on each other so the organization is competent and capable in adjusting to change. Each is necessary but insufficient on its own.

Continued on next page

[1] Could include system, community, organizational or individual levels.

[2] "Informed by" refers to earlier work that informed the development of the theory/framework.

[3] "Further development by" refers to new work evolving from the theory, often because it was an earlier framework.

[4] Components and dimensions are based on Zahra & George's (2002) reconceptualization of ACAP.

	1.) Field<br>2.) Type of theory/framework<br>3.) Level of focus	Focus of theory/framework	Approach to theory/framework development	Development or evolution of theory/framework	Core components and sub-components of theory/framework
Active Implementation Framework (Fixsen et al. 2005)	1.) Various<br>2.) Determinant framework<br>3.) Community, organizational, individual	To identify what influences the implementation of well-defined programs and practices.	Authors conducted a literature synthesis to determine what is known about relevant. components and conditions of implementation across various domains. Review methods were clearly outlined. Authors then discussed the results of the literature review and presented a conceptual framework.	Informed by:<br>- Kitson et al. (1998)	Implementation Framework Components:<br>1.)Source<br>2.)Destination<br>3.)Communication link between the source and destination<br>4.)Feedback mechanism at all levels<br>5.)Sphere of influence under which the other components operate<br>Core Implementation Components/Drivers:<br>1.) Staff selection<br>2.) Preservice and inservice training<br>3.) Ongoing consultation and coaching<br>4.) Staff and program evaluation<br>5.) Facilitative administrative support<br>Systems interventions
Consolidated Framework for Implementation Research (Damschroder et al. 2009)	1.) Health care<br>2.) Determinant framework<br>3.) Community, organizational, individual	To study and understand the implementation and associated constructs of new knowledge, tools, and practices across multiple levels of the healthcare system.	Authors used a snowball sampling approach to identify published theories, resulting in 19 frameworks and models from various fields and disciplines. Authors then evaluated the theories and assimilated and consolidated their constructs.	Informed by:<br>- Greenhalgh et al. (2004)<br>- Fixsen et al. (2005)<br>- Klein & Sorra (1996)<br>- Kitson (1997)	Framework illustrates an overarching taxonomy of factors that influence implementation and a way to organize and structure our observations.<br>Main Domains:<br>1.) Intervention characteristics (eight constructs)<br>2.) Outer setting (four constructs)<br>3.) Inner setting (12 constructs)<br>4.) Characteristics of the individuals involved (five constructs)<br>5.) The process of implementation (eight constructs).

Continued on next page

	1.) Field<br>2.) Type of theory/framework<br>3.) Level of focus	Focus of theory/framework	Approach to theory/framework development	Development or evolution of theory/framework	Core components and sub-components of theory/framework
Diffusion of Innovations for Service Organizations (Greenhalgh et al. 2004)	1.) Health service policy and management<br>2.) Determinant framework<br>3.) System, community, organizational, individual	To support the spread and sustainability of innovations in health service delivery and organizations through consideration of determinants, diffusion, dissemination and implementation.	Authors conducted a systematic review of the literature (meta-narrative review) across 13 research areas providing relevant evidence. Review methods were clearly outlined. They proposed a unifying conceptual model derived from the synthesis of the theoretical and empirical findings.	Informed by:<br>- Kitson et al. (1998)<br>- Zahra & George (2002)	Main Components:<br>1.) The innovation<br>2.) Adoption by individuals<br>3.) Assimilation by the system<br>4.) Diffusion and dissemination<br>5.) System antecedents for innovation<br>6.) System readiness for innovation<br>7.) The outer context: interorganizational networks and collaboration<br>8.) Implementation and routinization<br>9.) Linkage among components of the model<br>Each component includes various sub-factors.
Ecological Framework (Durlak & Dupre, 2008)	1.) Prevention and promotion<br>2.) Determinant framework<br>3.) Community, organizational, individual	To identify factors affecting the implementation process.	Authors conducted a literature review to identify the impact of implementation on program outcomes and the factors affecting the implementation process with a focus on prevention and health promotion programs for children and adolescents. Review methods were clearly outlined.	Informed by:<br>- Dane & Schneider (1998)<br>- Various others identified in literature review	The implementation process is affected by variables within five categories. Each category includes additional sub-factors. All five categories interact with each other and can lead to effective implementation.<br>Categories:<br>1.) Innovation characteristics<br>2.) Provider characteristics<br>3.) Community factors<br>4.) Prevention delivery system (i.e., organizational capacity and functioning)<br>5.) Prevention support system (i.e., training and technical assistance)

Continued on next page

	1.) Field<br>2.) Type of theory/framework<br>3.) Level of focus	Focus of theory/framework	Approach to theory/framework development	Development or evolution of theory/framework	Core components and sub-components of theory/framework
Implementation Effectiveness Model (Klein & Sorra, 1996)	1.) Management<br>2.) Implementation theory<br>3.) Organizational, individual	To examine the determinants of the effectiveness of organizational implementation.	Authors developed the model based on an understanding and non-systematic synthesis of the literature. Review methods were not described.	Further development by:<br>- Klein et al. (2001) further refined the model using empirical data from the manufacturing sector.<br>- Helrich et al. (2007) further refined the model using health care. Their proposed model is similar to that of Klein et al. (2001), but with the additional concept of innovation champions.	Implementation effectiveness is a function of:<br>1.) The strength of an organization's implementation climate for the given innovation (scale: strong vs weak)<br>2.) The fit of the innovation to targeted users' values (scale: good, neutral, poor)<br>Implementation climate is influenced by:<br>- Employees' skills in the use of the innovation<br>- Incentives to use the innovation and disincentives to not use the innovation<br>- Absence of obstacles in using the innovation<br>- Presence of innovation champions[5]
Multilevel Change Framework (Ferlie & Shortell, 2001)	1.) Quality improvement<br>2.) Determinant framework<br>3.) System, organizational, community	To present a multi-level approach to recognize the importance of core properties of successful quality-improvement work.	Authors examined quality strategies in the US and UK according to the multi-level approach and core properties. Authors highlighted factors that might influence adaptation and associated properties. Review methods were non-systematic and were not described.	Informed by:<br>- Goes et al. (2000)	Four Essential Core Properties:<br>1) Leadership at all levels<br>2) An organizational culture that supports learning throughout the care process<br>3) Cultivation of effective teams<br>4) Greater use of information technology for continuous improvement and external accountability<br>Framework Levels:<br>- Individual<br>- Group/team<br>- Overall organization<br>- Larger system/environment

Continued on next page

[5] Innovation champions was an addition to the model proposed by Helrich et al. (2007).

	1.) Field<br>2.) Type of theory/framework<br>3.) Level of focus	Focus of theory/framework	Approach to theory/framework development	Development or evolution of theory/framework	Core components and sub-components of theory/framework
Promoting Action on Research Implementation in Health Services (Kitson et al., 1998)	1.) Nursing<br>2.) Determinant framework<br>3.) Community, organizational, individual	To present successful research implementation as a function of the dynamic and simultaneous relationship between evidence, context, and facilitation.	Authors developed the theory from the collective experience gained from research, practice development, and quality improvement projects.	Informed by:<br>- Lomas (1994)<br>- Haines and Jones (1994)<br>- Department of Health in England (1996)<br>- Kitson et al. (1998)<br>Further development by:<br>- Rycroft-Malone (2002) provided more details on definitions of the PARIHS sub-elements.<br>- Kitson et al. (2008) outlined the PARIHS framework and how it could be used as a 'diagnostic and action tool' to evaluate evidence and context to create an appropriate facilitation strategy.<br>- Helfrich (2010) provided a thorough overview of the elements and sub-elements of the model.	Core Elements[6]:<br>1.) the level and nature of the evidence<br>2.) the context or environment into which the research is to be placed<br>3.) the method of facilitation<br>Sub-elements (high-to-low scale):<br>Evidence: research, clinical experience/expert opinion, patient experience/preferences client preferences, local data/information/information from local context/routine data.<br>Context: culture, leadership, measurement/evaluation, receptive context<br>Facilitation: characteristics, role, style, purpose, skills and attributes (expressed through an array of mechanisms)<br>Core elements are considered as three axes on a three dimensional matrix, such that a high score of any element would be expected to positively influence a successful implementation, while a low score would negatively affect implementation.

Continued on next page

[6] Core elements and sub-elements are a result of a compilation of the descriptions from Kitson et al. (1998), Rycroft-Malone (2002), Kitson et al. (2008), and Helfrich (2010).

	1.) Field<br>2.) Type of theory/framework<br>3.) Level of focus	Focus of theory/framework	Approach to theory/framework development	Development or evolution of theory/framework	Core components and sub-components of theory/framework
Sticky Knowledge (Szulanski, 1996)	1.) Management<br>2.) Implementation theory<br>3.) Community, organizational, individual	To investigate knowledge transfer within an organization and the associated internal stickiness (difficulties associated with knowledge transfer).	Author analyzed internal stickiness of knowledge transfer and tested the resulting model using analysis of a data set from eight companies.	Informed by:<br>- Prahalad and Hamel (1990)<br>- Grant (1991)	Origins of Stickiness:<br>1.) Knowledge (causal ambiguity*, unproven knowledge)<br>2.) Source (lacks motivation or perceived reliability)<br>3.) Recipient (lacks innovation, absorptive capacity*, or retentive capacity)<br>4.) Context (barren context, arduous relationship*)<br>* Identified as the most important knowledge-related barriers to internal knowledge transfer.<br>Stickiness can occur at different points along the transfer process. Problems are specific to different stages.<br>Process Stages:<br>- Initiation<br>- Implementation<br>- Ramp-up<br>- Integration.
Theoretical Domains Framework (Michie et al., 2005) & Behaviour Change Wheel (Michie et al., 2011)					TDF: 128 explanatory constructs were identified and grouped into domains.<br>TDF Domains[7]:<br>1.) Knowledge<br>2.) Skills<br>3.) Social/professional role and identity<br>4.) Beliefs about capabilities<br>5.) Optimism<br>6.) Beliefs about consequences<br>7.) Reinforcement<br>8.) Intentions<br>9.) Goals

Continued on next page

[7] Presented here are the 14 domains adapted by Cane et al. (2012) from Michie et al.'s (2005) original 12 domains.

	1.) Field<br>2.) Type of theory/framework<br>3.) Level of focus	Focus of theory/framework	Approach to theory/framework development	Development or evolution of theory/framework	Core components and sub-components of theory/framework
Theoretical Domains Framework (Michie et al., 2005) & Behaviour Change Wheel (Michie et al., 2011)	1.) Health psychology, health services<br>2.) Determinant framework<br>3.) Individual, system	TDF: To develop a comprehensive list of potential behavioural determinants.<br><br>BCW: To link behaviours to intervention functions and policies for evidence-based behaviour change intervention design.	TDF: Authors identified theoretical constructs, simplified them into construct domains, evaluated the importance of the construct domains; conducted an interdisciplinary evaluation, validated the domain list, and piloted interview questions.<br><br>BCW: Authors conducted a systematic search of electronic databases and consultation with behaviour change experts to identify and evaluate frameworks of behaviour change interventions. Review methods were described.	TDF informed by:<br><br>- Fishbein et al. (2001)<br>- Various other psychology theories<br><br>BCW informed by:<br><br>- The UK Medical Research Council, Institute for Government: MINDSPACE (2010)<br>- Cochrane Effective Practice and Organisation of Care Group, EPOC (2010).<br>- Michie et al. (2005) provided the early list of 12 domains (*not yet labelled as TDF*)<br>- Cane et al. (2012) expanded to 14 domains, resulting in the TDF<br>- Michie et al. (2011), developed the BCW in an effort to link the TDF to intervention design, (based on 19 behaviour change theories)	10.) Memory, attention and decision processes<br>11.) Environmental context and resources<br>12.) Social influences;<br>13.) Emotion<br>14.) Behavioural regulation<br><br>BCW: A key element of the behaviour system is the COM-B model:<br><br>- Capabilities<br>- Opportunity<br>- Motivation<br>COM-B is surrounded by nine intervention functions and seven policy categories to enable interventions.<br><br>BCW Intervention Functions:<br><br>1.) Education<br>2.) Persuasion<br>3.) Incentivization<br>4.) Coercion<br>5.) Training<br>6.) Restrictions<br>7.) Environmental restructuring<br>8.) Modelling<br>9.) Enablement<br><br>BCW Policy Categories:<br>1.) Communications/marketing<br>2.) Guidelines<br>3.) Fiscal measures<br>4.) Regulation<br>5.) Legislation<br>6.) Environmental/social planning<br>7.) Service provision
